# Radiological exploration on adjacent segments after total cervical disc replacement with Prodisc-C prosthesis

**DOI:** 10.1186/s13018-019-1194-x

**Published:** 2019-05-28

**Authors:** Shuai Xu, Yan Liang, Fanqi Meng, Kaifeng Wang, Haiying Liu

**Affiliations:** Department of Spinal Surgery, Peking University People’s Hospital, Peking University, No. 11 Xizhimen South Street, Xicheng District, Beijing, 100044 People’s Republic of China

**Keywords:** Total cervical disc replacement, Adjacent segments, Range of motion, Lordosis, Intervertebral disc height

## Abstract

**Purpose:**

The relationship between upper or lower adjacent segments (UAS/LAS) and the cervical spine parameters was not clear yet. So, the purpose was to analyze range of motion (ROM), lordosis (LOR), and intervertebral disc height (IDH) of UAS and LAS before and after total cervical disc replacement (TDR) and to explore the influencing factors of cervical spine radiological parameters on adjacent segments.

**Methods:**

A single-center retrospective study was performed on patients completing 10-year follow-up undergone TDR. As the primary outcomes, radiological parameters included UAS-ROM/LAS-ROM, UAS-LOR/LAS-LOR, and UAS-IDH/LAS-IDH. The secondary outcomes were ROM and LOR of C2–C7 and surgical levels, IDH of surgical segments, prosthesis migration, subsidence, heterotopic ossification (HO), and adjacent segment degeneration (ASD), which were measured on X-ray.

**Results:**

UAS-ROM and LAS-ROM remained stable in follow-up periods. There was no significance on UAS-LOR or LAS-LOR between pre- and post- operation, so was UAS-IDH or LAS-IDH. UAS-ROM was larger in the segments with ASD (*P* < 0.001), the same to LAS-ROM (*P* < 0.001), and UAS-LOR was larger in segments with ASD (*P* = 0.02). UAS-ROM was positively correlated with C2–C7 ROM and LOR (both *P* < 0.001). UAS-LOR was correlated with operated-segmental LOR while LAS-LOR were in correlation with surgical segment ROM. The influencing factors of UAS-ROM were the surgical segment ROM and C2–C7 LOR. The influencing factors of UAS-LOR and LAS-LOR were LAS-ROM and UAS-ROM, respectively. The influencing factors of UAS-IDH were LAS-IDH, surgical segment IDH, and HO while that of LAS-IDH were UAS-IDH and surgical segment IDH.

**Conclusions:**

TDR has only a little effect on the adjacent segments. There is an interaction between UAS and LAS. The maintenance on surgical segments ROM and reconstruction of IDH will benefit to adjacent segments.

## Introduction

Total cervical disc replacement (TDR) as an alternative to anterior cervical discectomy and fusion (ACDF) in the treatment of cervical disc degenerative disease (CDDD) has been widely accepted [1–3]. Numerous biomechanical experiments suggested TDR can theoretically preserve the activity of the surgical segment and reduce the stress of adjacent segments, which may reduce the occurrence of adjacent segment degeneration (ASD) [4, 5]. Although no consensus on the definition of ASD, there have been many publications supported the reduction of ASD after TDR compared with ACDF. Hilibrand et al. [6] believed that the range of motion (ROM), lordosis (LOR), and intervertebral disc height (IDH) of adjacent segments can affect ASD, while there were more focus on operated segments in previous studies rather than a systematic report on radiological parameters of upper and lower adjacent segments (UAS/LAS) after TDR.

Studies have shown a potential impact on UAS and LAS by radiological parameters of the global cervical spine, especially by the operated segments [7, 8]. Thus, the reconstruction of the cervical spine alignment through TDR surgery may improve parameters of UAS and LAS and then may reduce the incidence of ASD, but the relationship between UAS or LAS and the cervical spine parameters was not clear yet. The artificial disc of Prodisc-C (Synthes, Paoli, PA, USA), as one of the motion-preserving technique, is a constrained ball-in-trough articulation, where the vertical keels on upper and lower end plates, inserted into adjacent vertebrae, enable alignment correction and immediate fixation [2, 9]. Therefore, the purpose of this study was to analyze ROM, LOR, and IDH of UAS/LAS before and after TDR with Prodisc-C prosthesis and to explore the influencing factors of cervical spine radiological parameters on UAS and LAS.

## Materials and methods

### Study design

We performed a single-center retrospective study on all consecutive patients who have undergone TDR. All patients were operated by the same senior surgeon.

### Patient enrollment

The inclusion criteria were patients with (1) single- to three-level CDDD between C3/4 and C6/7 levels, (2) no response to conservative treatment for at least 6 months, and (3) TDR by Prodisc-C prosthesis. The exclusion criteria were patients with (1) ossification of the posterior longitudinal ligament; (2) instability of target level; (3) severe osteoporosis; (4) prior cervical spine surgery; and (5) malignancy, infection, and inflammation. All individual participants have signed informed consents.

### Radiological parameters of adjacent segments

Radiological evaluation included anteroposterior-lateral and flexion-extensionX-ray plain. The measurements contained (1) UAS-ROM and LAS-ROM, (2) UAS-LOR and LAS-LOR, and (3) UAS-IDH and LAS-IDH, which were defined as primary outcomes. ROM was measured on neutral and dynamic X-ray images and LOR was on neutral lateral X-ray images, where a positive angle indicated lordosis while a negative angle meant kyphosis. IDH was acquired by an average of anterior edge height, middle line height, and posterior edge height of the disc space on neutral lateral X-ray images [9].

The database was obtained preoperatively and the parameters were followed up at 1 week, 6 months, 1 year, 2 years, 5 years, 10 years, and the final visit after TDR in November 2018.

### Radiological parameters of the cervical spine

As secondary outcomes, we described on the following radiographs: (1) ROM of the whole cervical spine (C2–C7) and surgical levels, (2) LOR of C2–C7 and surgical levels, and (3) IDH of surgical levels. The complications were (1) implant migration (including coronal and sagittal migration), (2) implant subsidence, (3) heterotopic ossification (HO), and (4) ASD (including the cranial and caudal adjacent segments). Implant migration was defined by more than a 3-mm anteroposterior or coronal slip of the implant parallel to the vertebral endplates [2, 10]. Subsidence referred to bone penetration of the implant of more than 3 mm into the superior or inferior endplate of the adjacent vertebral body [2, 11]. HO can be classified according to the classification system of McAfee et al. [12] quantified from grade 0 (no HO present) to grade IV (complete fusion of the treated segment). ASD was defined by any presence of (1) enlarged ossification of the anterior longitudinal ligament, (2) an increased disc space narrowing > 30%, and (3) anterior enlarged osteophyte formation [3, 10]. Two independent orthopedic spine surgeons with > 5 years of experience in the field performed two series of primary and secondary parameter measurements.

### Statistical analysis

Paired *t* test was applied on comparison on pre- and post-operative primary outcomes, and independent sample *t* test was used on primary outcomes between the ASD group and non-ASD group. Pearson or Spearman correlation analysis was performed to explore the correlation among primary outcomes as well as between primary and secondary outcomes. Multiple linear regression analysis was performed to identify the influencing factors of primary outcomes. The statistical analysis was performed using IBM SPSS Statistics 22.0 and statistical significance was defined as *P* < 0.05.

## Results

### Patient enrollment

One hundred sixty patients who underwent TDR from March 2005 to September 2008 were included in this study. A total of 118 participants completed a minimum of 10 years follow-up (a mean of 135.75 months) with a follow-up rate of 73.8%. The sample consisted of 66 male and 52 female with a mean age of 46.84 ± 9.39 (years). Patients diagnosed with radiculopathy, myelopathy, and myeloradiculopathy were 39 cases, 27 cases, and 52 cases respectively, of whom there were 90 cases of single-level TDR, 20 cases of double-level TDR, and 8 cases of three-level TDR with an overall of 154 discs performed TDR. The most operated segments were C5/6 (90/154), and the main UAS/LAS were C6/7 (32.6%) and C4/5 (32.2%) (Table 1).

### Primary outcomes of adjacent segments

**Fig. 1:**
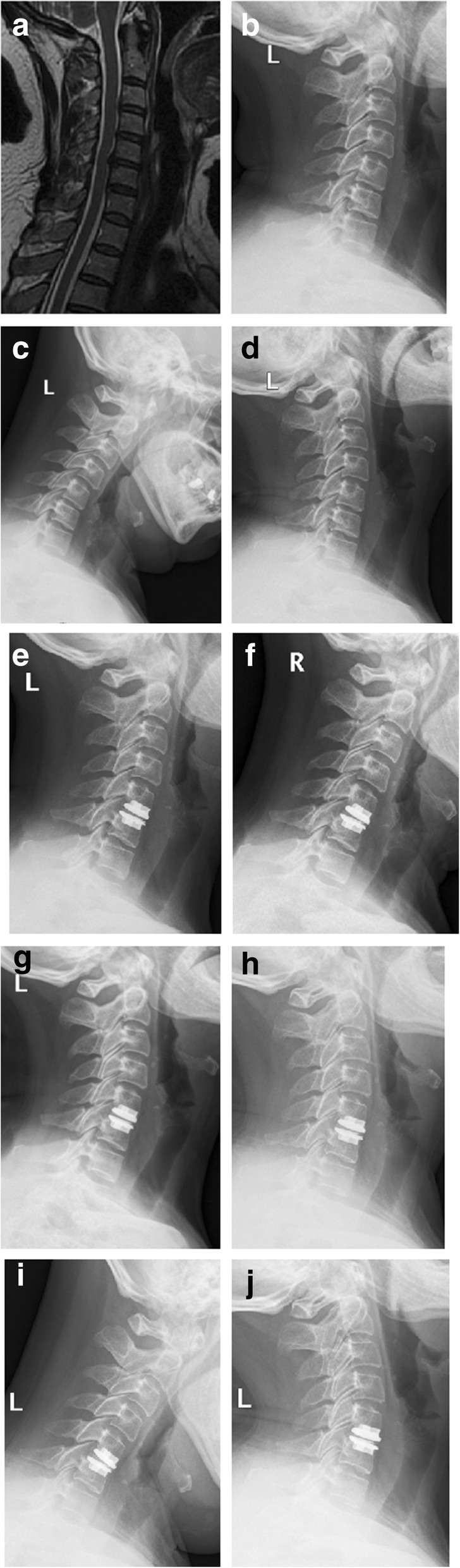
The primary outcomes before and after C5–6 TDR on a 54-year-old female. C4–5 is UAS and C6–7 is LAS. **a** C5-6 CDDD on the T2-weighted image of MRI before TDR; **b**–**d** Neutral lateral X-ray and flexion-extension X-ray before TDR. ROM, LOR, and IDH of C5–6 are 8.6°, − 2.3° and 3.76 mm, respectively; The primary outcomes of C4–5 and C6–7 are 9.7°, 8.5°, 4.41 mm and 5.7°, − 3.6°, 3.37 mm, respectively. **e**–**g** Neutral lateral X-ray and flexion-extension X-ray 1 week after TDR. ROM, LOR, and IDH of C5–6 are 7.3°, − 3.5°, and 6.85 mm, respectively; the primary outcomes of C4–5 and C6–7 are 6.3°, 4.8°, 4.19 mm and 4.8°, − 2.6°, 3.85 mm, respectively. **h**–**j** Neutral lateral X-ray and flexion-extension X-ray 10 years after TDR. ROM, LOR, and IDH of C5–6 are 9.0°, − 2.7°, and 6.55 mm, respectively; The primary outcomes of C4–5 and C6–7 are 8.1°, 7.0°, 4.61 mm and 6.5°, − 4.1°, 3.84 mm, respectively. There is no statistical change on primary outcomes of C4–5 and C6–7 although a higher IDH of C5–6 after TDR.

The mean UAS-ROM was 9.10° ± 5.78° preoperatively and 6.43° ± 4.40°, 7.96° ± 4.16°, and 7.69° ± 3.86° at 1 week, 1 year, and 10 years after surgery respectively. Mean LAS-ROM was 7.94° ± 5.95°, 6.57° ± 4.98°, 8.14° ± 5.76°, and 7.41° ± 4.31° respectively at the same follow-up periods. UAS-ROM and LAS-ROM kept stable at all follow-up periods except for a lighter decrease in UAS-ROM 1 week after surgery. Mean UAS-LOR and LAS-LOR were 3.28° ± 5.82°, 2.28° ± 5.36°, 3.75° ± 3.46° and 3.91° ± 5.32°, 2.94° ± 5.21°, 5.03 ± 4.95° respectively before surgery and after surgery at 1 week and 10 years. There was no statistical difference between the baseline and all postoperative follow-up. Mean UAS-IDH (mm) were 4.72 ± 0.6, 4.76 ± 0.71, 4.66 ± 0.71, and 4.71 ± 0.72 respectively before surgery, 1 week, 1 year, and 10 years after surgery; the mean LAS-IDH (mm) were 4.73 ± 0.95, 4.95 ± 1.10, 4.78 ± 1.01, and 4.64 ± 0.95, respectively. There were no statistical differences between preoperative and all postoperative periods both in UAS-IDH and LAS-IDH (*P* = 0.875 and *P* = 0.833, respectively) (Table 2, Fig. 1).

**Fig. 2:**
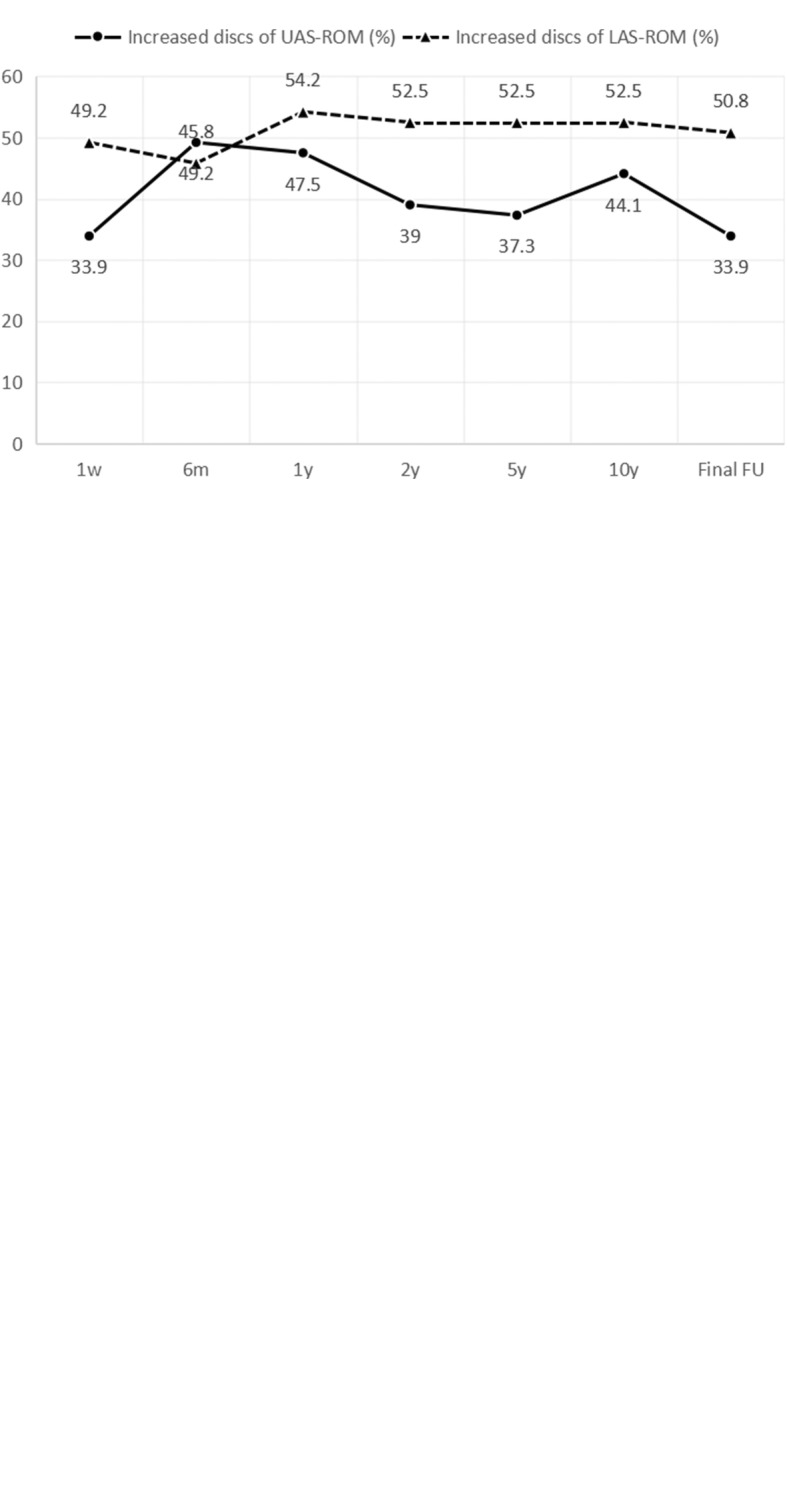
The proportion of modified discs post-operatively on primary outcomes at every FU period. **a** The proportion of the number of segments acquiring increased UAS-ROM and LAS-ROM at every FU period. **b** The proportion of positive lordosis of the upper and lower adjacent segment at every FU period. **c** The proportion of UAS-IDH and LAS-IDH increasing segments at every FU period (FU follow up, UAS-ROM range of motion of upper adjacent segment, LAS-ROM range of motion of lower adjacent segment, UAS-LOR lordosis of upper adjacent segment, LAS-LOR lordosis of lower adjacent segment, UAS-IDH intervertebral disc height of upper adjacent segment, LAS-IDH intervertebral disc height of lower adjacent segment)

The proportion of increased discs on UAS-ROM was 33.9–49.2% while decreased segments on UAS-ROM was 50.8–66.1% after TDR and that of increased segments on LAS-ROM was 45.8–54.2%. At all postoperative follow-up, the positive lordosis of UAS-LOR and LAS-LOR were 66.7–85.0% and 66.7–88.9% respectively. Compared with baseline, the ratio of increased segments on UAS-IDH and LAS-IDH were 37.3–55.9% and 35.6–67.8% respectively (Fig. 2).

### Primary outcomes with ASD and non-ASD

Two of 154 cases of UAS-ASD occurred at 1 week after surgery with a gradual increase from 1 year (5.0%) to 10 years (21.8%) after surgery (*P* < 0.001); LAS-ASDappeared in four levels 1 week later and it gradually increased from 1 year (7.6%) to the final follow-up (20.2%) (*P* < 0.05).

**Fig. 3:**
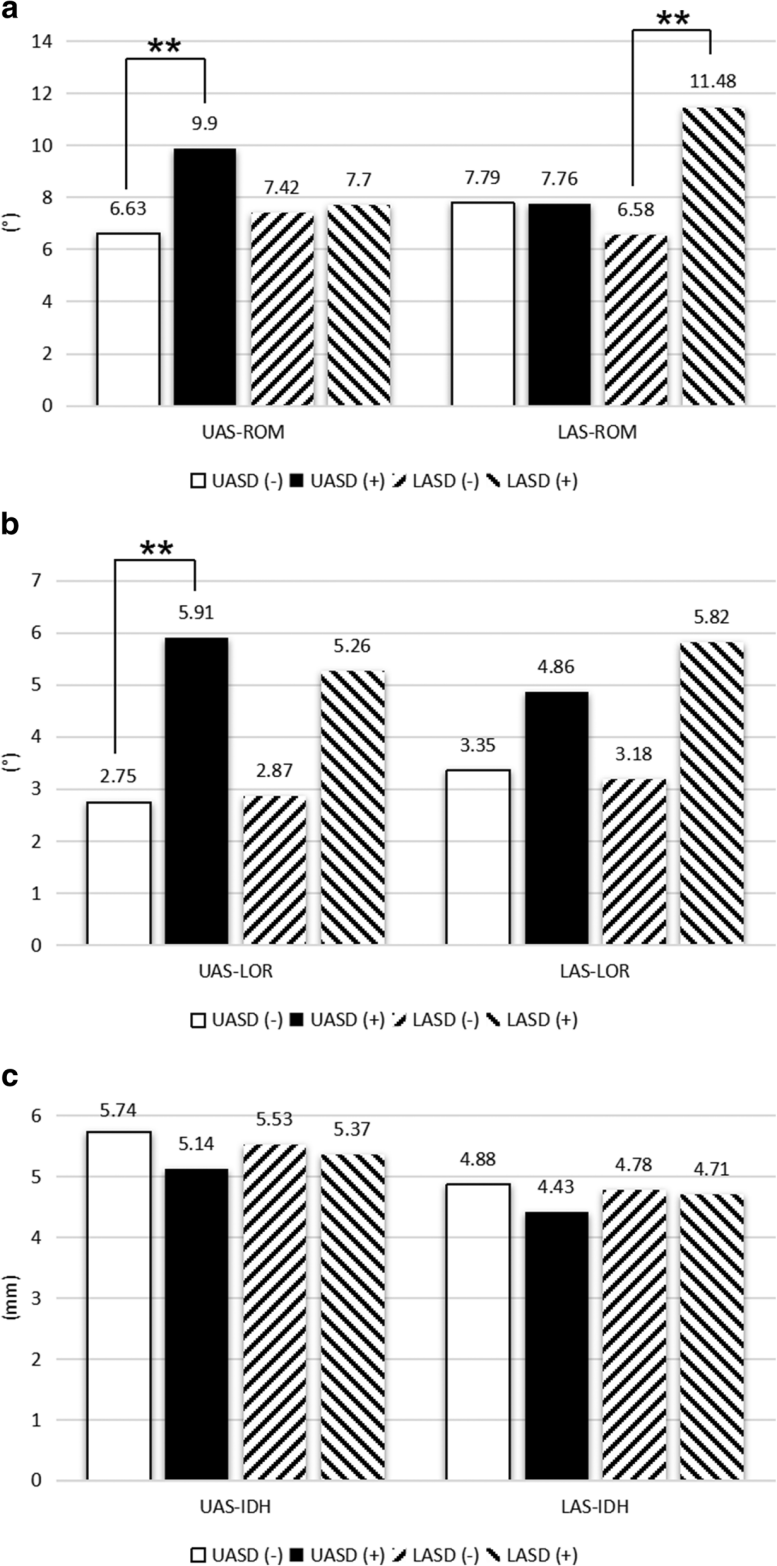
Comparison of primary outcomes between the ASD group and non-ASD group. **a** Comparison of UAS-ROM and LAS-ROM between the ASD group and non-ASD group. **b** Comparison of UAS-LOR and LAS-LOR between the ASD group and non-ASD group. **c** Comparison of UAS-IDH and LAS-IDH between the ASD group and non-ASD group (ASD upper adjacent segment degeneration, UAS-ROM range of motion of upper adjacent segment, LAS-ROM range of motion of lower adjacent segment, UAS-LOR lordosis of upper adjacent segment, LAS-LOR lordosis of lower adjacent segment, UAS-IDH intervertebral disc height of upper adjacent segment, LAS-IDH intervertebral disc height of lower adjacent segment, UASD upper adjacent segment degeneration, LASD lower adjacent segment degeneration, *P < 0.05 of intergroup comparison, **P < 0.01 of intergroup comparison)

Figure 3 showed that UAS-ROM in UAS-ASD cases was larger than those without ASD (*P* < 0.001), so was LAS-ROM in LAS-ASD group (*P* < 0.001). UAS-LOR with UAS-ASD was larger than that of the non-ASD group (*P* = 0.02). The mean LAS-LOR with LAS-ASD was higher than that without ASD but there was no statistical difference (*P* = 0.07). UAS-IDH and LAS-IDH both showed no difference between ASD and non-ASD group (*P* > 0.05).

### Correlation among primary outcomes

UAS-ROM was positively correlated with LAS-ROM (*P* < 0.001), and LAS-ROM was also positively correlated to LAS-IDH.UAS-LOR was in positive correlation with LAS-LOR (*P* < 0.001) and LAS-IDH while LAS-LOR was positively correlated with UAS-IDH and LAS-IDH.UAS-IDH was positively correlated with LAS-IDH (*P* < 0.001) (Table 3).

### Correlation between primary and secondary outcomes

UAS-ROM was positively correlated with C2–C7 ROM (*P* < 0.001), C2–C7 LOR (*P* < 0.001), and ROM of surgical segments (*P* = 0.005) while LAS-ROM was in correlation with C2–C7 ROM and surgical segment ROM. UAS-LOR was positively correlated with surgical segment LOR (*P* = 0.014) and LAS-LOR was positively correlated with surgical segment ROM (*P* = 0.048). LAS-IDH was positively correlated with ROM and IDH of surgical segments (both *P* < 0.01) while was negatively correlated with HO (*P* < 0.001) (Table 4).

### Multiple linear regression analysis on primary outcomes

Multiple linear regression analysis on primary outcomes showed that the influencing factors of UAS-ROM were surgical segment ROM (*P* < 0.001) and C2–C7 LOR (*P* = 0.015) while that of LAS-ROM were surgical segment ROM and HO (both *P* < 0.001). The influencing factors of UAS-LOR and LAS-LOR were LAS-LOR and UAS-LOR, respectively (both *P* < 0.001). The influencing factors of UAS-IDH were LAS-IDH (*P* < 0.001), surgical segment IDH (*P* = 0.002), and HO (*P* = 0.015) and that of LAS-IDH were UAS-IDH (*P* = 0.001) and surgical segment IDH (*P* = 0.045) (Table 5).

## Discussion

Studies have shown that the incidence of ASD at 10-year follow up after ACDF, the classic procedure for the treatment of CDDD, is as high as 25–90% [10]. ASD was considered to be linked with the loss of motion, the change of movement of adjacent segments, and intervertebral disc stress [13]. The load of adjacent segments will change due to the prosthesis implantation and facet fusion while non-fusion technique with dynamic stabilization brought could restore spine stability and reduce the stress shelter effect [14]. Barrey et al. [15] reported that TDR put no significant effect on the motion of the cervical spine and the pressure of adjacent segments. Chang et al. [7, 16] compared cervical spine ROM after ACDF and TDR by computer-assisted vertebral motion analysis and found TDR can preserve more ROM of index segments ROM and can reduce UAS-ROM and LAS-ROM, verifying the hypothesis that TDR may reduce the occurrence of ASD by preventing excessive ROM of adjacent segments. This study firstly confirmed that TDR had little effect on adjacent segments. In addition, it remained that spinal surgeons should know what they need to do and which parameters they should focus on for the safety of adjacent segments.

Park et al. [17] completed a 5-yearfollow-up of 21 patients with ACDF, showing that the C2–C7 ROM was the same as the baseline while increased on adjacent segments. Tian et al. [18] found no significant changes on UAS-ROM and LAS-ROM but a reduction on C2–C7 ROM after ACDF. Chang et al. [7] found that LAS-ROM increased after TDR, such as an increased ROM on C6/7 after C4/5-operated TDR. They considered the possible stiff neck caused by preoperative pain limited the activity and postoperative pain relief as well as paraspinal muscle relaxation allowed more ROM. The study found a reduction of UAS-ROM 1 week after surgery probably resulted from the limitations of the cervical collar and the psychological effect of the patient. During one decade after surgery, there were still more adjacent segments with increased ROM although nearly half of the cases suffered ROM reduction. In general, UAS-ROM and LAS-ROM remained stable after TDR.

Yu et al. [19] considered that non-operated segments increased ROM in order to maintain physiological activities after ACDF, where ROM gradually decreased cranially and caudally with a center of the operated segments. In this condition, ASD was incidental with adjacent segments overloaded. Yang et al. [20] measured the ROM and found 5.8 ° ± 1.6 ° and 8.3 ° ± 2.7 ° in the ASD group and non-ASD group respectively on the surgical segments. They also found that the surgical segment ROM was not a risk factor for ASD. In this study, the comparison of adjacent segment ROM in the ASD and non-ASD group showed a significant increase in the ASD group, which was indirectly consistent with Yang’s study. However, there was a lack of strength in the evidence yet due to the heterogeneity on ASD definition [21].

UAS-LOR and LAS-LOR remained stable after TDR in our study, considering little effect on the local adjacent segment sequence after the prosthesis implantation. The normal cervical spine alignment was important for maintaining the biomechanical environment, which could be impaired by unsuitable fixation, prosthesis subsidence, and implant loosening then lead to ASD [22]. Hwang et al. [23] found that increased C2–C7 LOR would reduce the adjacent segment ROM and relief the incidence of adjacent disc degeneration. The straight or kyphotic cervical spine were more likely to suffer symptoms than normal ones by 18 times and neck pain was significantly associated with LOR [24]. It was concluded that the adjacent segment LOR in the ASD group was larger than that of the non-ASD group in our study, suggesting excessive adjacent segmental LOR may cause ASD.

Once with the loss of LOR on index and adjacent segments, the moment of the instantaneous rotation axis would increase when the cervical vertebrae were put under axial load, causing disorder of original load balance [8]. In addition, the loss of cervical spine alignment could also cause intervertebral foramen stenosis, relaxation, and wrinkling of the ligament in spinal canal and reduction of the anteroposterior diameter of the spinal canal, which usually involved adjacent segments and consequently caused radiculopathy and myelopathy [25].

TDR can maintain disc pressure of the surgical and adjacent segments. Laxer et al. [26] simulated the flexion-extension movement of double-level TDR and ACDF under load conditions on cadaver specimens, determining a higher adjacent segment disc pressure after ACDF and a correlation between intervertebral disc pressure and IDH. Li et al. [27] found a preoperative IDH of 3.5 ± 1.3 mm in the ASD group and 4.9 ± 1.2 mm in the control group, while the postoperative IDH was 9.6 ± 1.4 mm in the ASD group and 7.1 ± 1.2 mm in the control group in 116 cases of ACDF. Postoperative IDH was the main factor for accelerating ASD, and according to Laxer's theory, intervertebral pressure and IDH remained stable after TDR and the stress distribution in the three adjacent segments changed little contrasted with baseline.

Our results suggested an interaction between UAS and LAS. Firstly, the biomechanical mode on adjacent segments had a displacement control mode and moment control mode. Jiang et al. [28] proposed a ROM redistribution theory for each segment to obtain the original C2–C7 ROM after surgery based on the hybrid of the two modes. By which, the interaction between UAS and LAS promoted to achieve the balance for more suitable postoperative activity. Secondly, intervertebral disc and two facets were three important structures for balancing the movement between the vertebral bodies. The instability and degeneration of any structure would affect the motion quality. The surgical segment, as the most severe level by natural degeneration and iatrogenic interference, interacted with the functional spinal units composed of UAS and LAS [15, 29]. Faizan et al. [30] confirmed that ACDF would affect the inter-facet pressure of adjacent segments but less after TDR. Thirdly, the prosthesis increased the stiffness of index segments and then the load between adjacent segments was transmitted to each other through the prosthesis, causing a mutual increased loads of adjacent segments [8]. TDR could mimic the anatomical features of the intervertebral disc and effectively maintain the disc pressure within a normal range, which could explain the reduction of ASD occurrence with TDR[31].

The results of this study may provide some advice for operation. In addition to the biomechanical differences between ACDF and TDR reported in previous studies, surgical technique would have an impact on the results. Tu et al. [32] performed a 2-yearfollow-up in 107 patients with TDR and found that surgical skill affected ASD occurrence and the ROM of surgical segments, while all the patients were operated by the same surgeon in our study. Other than lesions removal, it was worthy of mobility maintaining, proper intervertebral space distraction, and cervical alignment recovery. Ishihara et al. [33] proposed that excessive removal of vertebrae during TDR implantation can lead to ossification of the anterior border and may favor ASD occurrence. Hwang et al. [23] also confirmed that TDR had a high impact on the sagittal motion by different types of test models. It was reported that TDR would change towards to the biomechanics of ACDF with gradual deterioration with HO in the intervertebral space [34]. Therefore, it was necessary to minimize the incidence of iatrogenic HO.

There are limitations in our study. Firstly, there is a lack of a control group and no comparison of adjacent segments of patients, and the conclusion will be more reliable where there is a comparison between ACDF and TDR. In addition, radiograph interpretation can lead to measurement bias, because cervical movement is multidirectional whereas ROM or IDH in only one plane.

## Conclusions

A 10-yearfollow-up of Prodisc-C showed that TDR had only a little influence on adjacent segments. There is an interaction on UAS and LAS. Keeping the ROM of surgical segments, recovery of IDH, and reduction of HO will bring benefit on adjacent segments and reduce the incidence of ASD.

## Data Availability

The datasets used and/or analysed during the current study are available from the corresponding author on reasonable request.

## Abbreviations

ACDF: Anterior cervical discectomy and fusion
ASD: Adjacent segment degeneration
CDDD: Cervical disc degenerative disease
HO: Heterotopic ossification
IDH: Intervertebral disc height
LAS: Lower adjacent segments
LOR: Lordosis
ROM: Range of motion
TDR: Total cervical disc replacement
UAS: Upper adjacent segments
