# Correction to: Radiological exploration on adjacent segments after total cervical disc replacement with Prodisc-C prosthesis

**DOI:** 10.1186/s13018-019-1249-z

**Published:** 2019-07-08

**Authors:** Shuai Xu, Yan Liang, Fanqi Meng, Kaifeng Wang, Haiying Liu

**Affiliations:** Department of Spinal Surgery, Peking University People’s Hospital, Peking University, No. 11 Xizhimen South Street, Xicheng District, Beijing, 100044 People’s Republic of China

**Correction to: J Orthop Surg Res (2019) 14:160**

**https://doi.org/10.1186/s13018-019-1194-x**

In the original publication of this article [1], the Table 1, 2, 3, 4 and 5 were missing due to a mistake in the copy-editing stage.

Table 1, 2, 3, 4 and 5 are listed below:

**Table 1:** Demographic characteristics and CDDD information of patients

Parameters	Statistics
No. of patients	118
Gender	
Male	65 (55.1%)
Female	53 (44.9%)
Age (years)	46.85 ± 9.38
BMI (kg/m2)	24.62 ± 3.72
Follow-up (months)	135.75 ± 8.46
Diagnosis	
Radiculopathy	39 (33.0%)
Myelopathy	27 (22.9%)
Radiculopathy & Myelopathy	52 (44.1%)
No. of levels	
Single level	90 (76.3%)
Double levels	20 (16.9%)
Three levels	8 (6.78%)
Distribution of operated levels	
C3/4	10 (6.5%)
C4/5	32 (20.8%)
C5/6	90 (58.4%)
C6/7	22 (14.3%)
Distribution of adjacent segments	
C2/3	10 (4.2%)
C3/4	30 (12.7%)
C4/5	76 (32.2%)
C5/6	21 (8.9%)
C6/7	77 (32.6%)
C7/T1	22 (9.3%)

Footnote: *CDDD* Cervical disc degeneration disease, *No.* Number, *BMI* Body mass index

**Table 2:** Radiographic primary outcomes from preoperation to the final FU

	Preoperation	1-week FU	6-month FU	1-year FU	2-year FU	5-year FU	10-year FU	Final FU
ROM (°)								
UAS	9.10 ± 5.78	6.43 ± 4.40**	7.83 ± 5.36	7.96 ± 4.16*	7.72 ± 4.97	7.60 ± 4.44	7.59 ± 3.86	7.38 ± 3.55*
LAS	7.94 ± 5.95	6.57 ± 4.98	7.61 ± 5.32	8.14 ± 5.76	8.61 ± 5.61*	8.45 ± 5.16	7.41 ± 4.31	7.71 ± 4.33
LOR (°)								
UAS	3.28 ± 5.82	2.28 ± 5.36	2.80 ± 5.00	3.64 ± 4.97	4.75 ± 7.34	5.84 ± 5.50	3.75 ± 3.46	
LAS	3.91 ± 5.32	2.94 ± 5.21	3.94 ± 5.66	4.15 ± 5.15	3.16 ± 5.64	3.98 ± 4.03	5.03 ± 4.95	
IDH (mm)								
UAS	4.72 ± 0.66	4.76 ± 0.71	4.71 ± 0.74	4.66 ± 0.71	4.69 ± 0.72	4.71 ± 0.70	4.71 ± 0.72	4.69 ± 0.72
LAS	4.73 ± 0.95	4.95 ± 1.10	4.77 ± 0.96	4.78 ± 1.01	4.83 ± 0.90	4.73 ± 1.06	4.64 ± 0.95	4.66 ± 0.96

Footnote: *FU* Follow up, *ROM* Range of motion, *UAS* Upper adjacent segment, *LAS* Lower adjacent segment, *LOR* Lordosis, *IDH* Intervertebral disc height

*: Significance (P<0.05) in comparison to baseline

**: Significance (P<0.01) in comparison to baseline

**Table 3:** Pearson correlation analysis among primary outcomes

		UAS-ROM	LAS-ROM	UAS-LOR	LAS-LOR	UAS-IDH	LAS-IDH
UAS-ROM	r	1	0.206	0.043	0.014	0.018	0.011
	*P* Value		0.000	0.547	0.857	0.692	0.814
LAS-ROM	r		1	0.085	0.007	−0.054	0.104
	*P* Value			0.236	0.933	0.245	0.025
UAS-LOR	r			1	0.362	0.025	0.114
	*P* Value				0.000	0.727	0.113
LAS-LOR	r				1	0.115	0.154
	*P* Value					0.148	0.052
UAS-IDH	r					1	0.451
	*P* Value						0.000

Footnote: *UAS* Upper adjacent segment, *ROM* Range of motion, *LAS* Lower adjacent segment, *LOR* Lordosis, *IDH* Intervertebral disc height

**Table 4:** Correlation analysis between primary outcomes and secondary outcomes

		C2-C7 ROM	OP-ROM	C2-C7 LOR	OP-LOR	OP-IDH	Subsidence	Cor-M	Sag-M	HO
UAS-ROM	r	0.573	0.465	0.128	−0.043	− 0.075	− 0.027	− 0.041	0.095	− 0.068
	*P* Value	0.000	0.000	0.005	0.506	0.102	0.589	0.408	0.054	0.167
LAS-ROM	r	0.513	0.395	0.069	0.024	0.003	−0.060	0.028	0.052	0.128
	*P* Value	0.000	0.000	0.140	0.706	0.951	0.226	0.573	0.295	0.010
UAS-LOR	r	0.086	0.117	0.096	0.179	−0.038	0.183	0.124	0.086	0.072
	*P* Value	0.237	0.108	0.188	0.014	0.603	0.033	0.146	0.313	0.404
LAS-LOR	r	0.052	0.160	0.057	0.085	0.027	0.061	0.051	−0.001	− 0.029
	*P* Value	0.524	0.048	0.483	0.294	0.743	0.522	0.593	0.991	0.758
UAS-IDH	r	0.011	0.032	−0.029	0.005	0.070	0.018	0.042	0.043	−0.071
	*P* Value	0.804	0.484	0.526	0.942	0.129	0.718	0.397	0.379	0.152
LAS-IDH	r	0.057	0.131	0.089	0.058	0.179	0.150	0.019	−0.033	−0.173
	*P* Value	0.214	0.004	0.053	0.373	0.000	0.002	0.696	0.500	0.000

Footnote: *ROM* Range of motion, *OP* Operation segment, *LOR* Lordosis, *IDH* Intervertebral disc height, *Cor-M* Coronal migration, *Sag-M* Sagittal migration, *HO* Heterotopic ossification, *UAS* Upper adjacent segment, *LAS* Lower adjacent segment

**Table 5:** Multiple linear regression analysis of primary outcomes

	Coefficient	Unstandardized		Standardized	T	*P* value
		B	SE	Beta		
UAS-ROM	OP-ROM	0.603	0.052	0.499	11.702	0.000
	C2-C7 LOR	0.045	0.018	0.108	2.452	0.015
	OP-IDH	−0.009	0.158	− 0.002	− 0.054	0.957
	Sag-M	0.875	0.616	0.062	1.421	0.156
	HO	−0.283	0.173	−0.073	−1.635	0.103
LAS-ROM	LAS-IDH	0.417	0.232	0.082	1.799	0.073
	OP-ROM	0.606	0.062	0.435	9.802	0.000
	C2-C7 LOR	−0.010	0.022	−0.021	− 0.455	0.650
	HO	0.758	0.210	0.169	3.608	0.000
UAS-LOR	LAS-LOR	0.376	0.089	0.371	4.204	0.000
	LAS-IDH	−0.066	0.461	−0.013	− 0.143	0.887
	OP-ROM	0.260	0.143	0.159	1.811	0.073
	C2-C7 LOR	−0.013	0.045	−0.026	− 0.287	0.775
	OP-LOR	0.136	0.085	0.142	1.593	0.114
	Subsidence	4.124	2.283	0.182	1.806	0.074
	Cor-M	1.780	2.399	0.069	0.742	0.460
LAS-LOR	UAS-LOR	0.322	0.073	0.338	4.416	0.000
	UAS-IDH	0.418	0.631	0.055	0.662	0.509
	LAS-IDH	0.383	0.433	0.075	0.885	0.378
	OP-ROM	0.126	0.102	0.095	1.239	0.217
UAS-IDH	LAS-LOR	0.001	0.011	0.004	0.053	0.958
	LAS-IDH	0.225	0.062	0.322	3.606	0.000
	OP-IDH	0.138	0.043	0.297	3.206	0.002
	HO	−0.160	0.065	−.215	−2.475	0.015
LAS-IDH	LAS-ROM	0.012	0.021	0.054	0.539	0.591
	UAS-LOR	0.010	0.019	0.052	0.520	0.604
	LAS-LOR	0.005	0.019	0.028	0.284	0.777
	UAS-IDH	0.502	0.142	0.350	3.531	0.001
	OP-ROM	0.006	0.030	0.020	0.204	0.839
	C2-C7 LOR	0.000	0.009	−0.005	−0.055	0.957
	OP-IDH	0.141	0.070	0.213	2.030	0.045
	Subsidence	0.178	0.449	0.041	0.397	0.692
	HO	0.022	0.107	0.021	0.203	0.839

Footnote: *SE* Standard error, *UAS* Upper adjacent segment, *ROM* Range of motion, *LAS* Lower adjacent segment, *LOR* Lordosis, *IDH* Intervertebral disc height, *OP* Operation segment, *Sag-M* Sagittal migration, *HO* Heterotopic ossification, *Cor-M* Coronal migration

The original article has been corrected.
